# Comparison Between in Vitro Effects of Aqueous Extract of *Artemisia seiberi* and Artemisinin on *Leishmania major*

**Published:** 2013-05-04

**Authors:** Farzad Esavand Heydari, Fatemeh Ghaffarifar, Saied Soflaei, Abdolhosein Dalimi

**Affiliations:** 1 Department of Parasitology, Faculty of Medical Sciences, Tarbiat Modares University, Tehran, IR Iran

**Keywords:** Artemisinin, *Leishmania major*, *In Vitro*

## Abstract

**Background:**

It is necessary to develop novel, affordable, and accessible drugs with few side effects as alternatives of the currently available chemical agents for leishmaniasis.

**Objectives:**

The main purpose of this study was to evaluate the effects of these drugs on L. major under in vitro conditions.

**Materials and Methods:**

In the current study, 5, 10, 25, 50, and 100 µg/mL concentrations of aqueous extract of Artemisia sieberi and chemical artemisinin were tested on promastigotes of *Leishmania major* (*L. major*), uninfected macrophages, and infected macrophages with intracellular amastigotes of *L. major*, by direct counting and 3-(4 5-dimethylthiazol-2-yl)-2 5-diphenyltetrazolium bromid methods.

**Results:**

The results obtained for each drug were compared with other drugs and also with the results of the control groups. The results related to promastigote and amastigote assays showed that when the dose of both drugs increased, the parasite number is reduced in comparison with the control groups. Moreover, the parasitic burden in the test cultures decreased significantly. Macrophage assay results showed that the effects of both drugs on uninfected and healthy macrophages were very low.

**Conclusions:**

These results indicate that both drugs have anti-Leishmania effects, which was higher in *Artemisia sieberi* compared with artemisinin. Thus, carrying out further studies on the effects of *Artemisia sieberi* in infected animals with *L. major* is recommended.

## 1. Background

An important parasitic disease is leishmaniasis, which is still a widespread disease. Furthermore, preventing drug resistance is of great importance, and plant medicines may play a major role in reducing resistance to chemical drugs. This fact is considered by the World Health Organization (WHO) (1). New useful treatment or prevention strategies are needed. Using medicinal plants for treatment of diseases has a long history and people use these drugs since many years ago. Considering treatment of cutaneous leishmaniasis, most currently available drugs are of chemical origin, which are not useful enough to be the ultimate strategy in leishmaniasis treatment. It is estimated that one third of all drug agents are of plant origin or have been changed after extraction from plants (1). The WHO recommends artemisinin combination therapies as the first-line treatment for malaria (2, 3). Artemisinin and aqueous extract of *Artemisia sieberi* are of plant origin. Artemisinin is derived from a medicinal herb called *qinghao* (sweet wormwood) or Artemisia annua and is still obtained from this plant. Artemisinin is comparatively easily purified after extraction from plants. Artemisinin is a useful anti-malarial for many reasons and is one of the few classes of drugs useful to treat severe malaria resistant to chloroquine. It acts more rapidly than other types of antimalarial agents; both in killing the parasite and inhibition of parasite maturation (4). Considering the diversity in the climate condition and the diverse flora of Iran, identification of effective plant components in various native plants of the country and large scale extraction of the components is possible (5). *Artemisia sieberi* is a well-known medicinal plant that has been used in traditional medicine of the Middle East countries as a herbal medicine for treating various diseases (6). In Iran, the plant grows in the foothills of Alborz Mountain, Semnan, and south of the Iran plateau. *Artemisia sieberi* and artemisinin have the same origin; *qinghao* or Artemisia.

## 2. Objectives

The main purpose of this study was to evaluate the effects of these drugs on *L. major* under *in vitro* conditions.

## 3. Materials and Methods

### 3.1. Preparation of Aqueous Extract of *Artemisia sieberi*

The plant was collected from the foothills of Alborz and the Southern Iranian plateau. After cleaning, the aerial parts and roots were dried in shadow, powdered, and kept under appropriate conditions. To prepare aqueous extract, distilled water (10 mL per gram of the powder) was added to the powder and the mixture was boiled for 15 minutes. The filtered liquid was transferred to a solvent removal instrument; so 80% of its water was removed. Then the remainder water was removed by placing the sample in a warm bath at 30˚C. After filtration, 1 mL of the aqueous extract of *Artemisia sieberi* was incubated at 60˚C for 24 hours and the dry weight of drug in solution was determined.

### 3.2. Preparation of Artemisinin

Artemisinin (C_15_H_22_O_5_) was purchased from Enzo Life Sciences Company (Germany; batch number L16705) and prepared according to manufacturer’s protocol.

### 3.3. Cultured Parasites

The *L. major* (MRHO/IR/75/ER) promastigotes were cultured in Novy – MacNeal - Nicolle and then in complete RPMI1640 supplemented with 10% heat-inactivated fetal calf serum (Hi - FCS), 100 IU/mL of penicillin, and 100 μg/mL of streptomycin, and then incubated at 24 °C. For parasite proliferation, these cultures were weekly passaged and microscopically monitored.

### 3.4. Anti-promastigote Activity

Anti-promastigote assays were carried out using direct counting assay based on growth inhibition. Stationary phase of promastigotes (2 × 10^6^ promastigotes/mL) were harvested in complete RPMI. Then 100 µL of promastigote sample was transferred to 24-well plates containing 100 µL of complete RPMI and treated with serial concentrations of *Artemisia sieberi* and Artemisinin (5, 10, 25, 50, and 100 µg/mL) as test groups, without renewing the medium or drug, and incubated for 24, 48, and 72 hours. The parasites were counted by a light microscope and were compared with the control cultures. Each test was performed in triplicate and repeated in separate experiments.

### 3.5. Colorimetric MTT Method

The ability of cells in transforming yellow tetrazolium crystals to insoluble blue foromasan was evaluated by MTT colorimetric method (7, 8). This assay was employed for verification of promastigote assay results as described above. Briefly, 100 µL of promastigotes (2 × 10^6^ cells/mL) was added to 96-well plates containing 100 µL of complete RPMI medium supplemented with 10% fetal calf serum (FCS). These cultures were repeated in triplicate wells. Moreover, 200 µL of promastigotes were cultured as the control group. Then 200 µL/well of PBS was added around plate wells to prevent evaporation of well contents. The test groups were incubated in the presence of five concentrations of *Artemisia sieberi* and artemisinin at 24 ± 1°C for 72 hours, and then 20 µL of MTT solution was added to each well. Plates were incubated again at 24°C for 4 hours and then centrifuged at 1000 rpm for 10 minutes. Supernatant was aspirated gently and discarded. After that 100 µL DMSO was added to each well. The amount of color produced is directly relative to the number of viable cells. Relative numbers of living cells were determined based on the optical absorbance of the treated and untreated samples and blank wells, using the following formula: % viable cells = (absorbance of treated cells / absorbance of control cells) × 100.

### 3.6. Macrophage Cytotoxicity

For evaluation of the cytotoxic effect of *Artemisia sieberi* and artemisinin on uninfected mouse macrophages, we used inbred male BALB/c mice that were purchased from the Animal Lab of Razi Institute of Iran. The peritoneal macrophages were collected and the number of living macrophages was estimated. Briefly, 6 mL of RPMI medium (Sigma) was injected into mouse peritoneum and macrophages were collected and stained with trypan blue for estimation of living macrophages. Then, 100 µL of macrophages with 100 µL of RPMI1640 medium were seeded in the presence of five concentrations (5, 10, 25, 50, and 100 µg/mL) of *Artemisia sieberi* and artemisinin. These cultures were maintained at 37 ºC in the presence of 5% CO_2_ for 24, 48, and 72 hours. After this period of time, the experiment was terminated and cytotoxic effect of these drugs on uninfected macrophage was evaluated using direct counting. Moreover, the drugs were compared with each other and with the control cultures in this regard.

### 3.7. Anti-amastigote Activity

Peritoneal macrophages were extracted from the peritoneal cavity of BALB/c mice. Afterwards, isolated macrophages were seeded in 24-well plates and incubated at 37 °C with 5% CO_2_ for 24 hours for differentiation of macrophages. Adherent macrophages were infected with promastigotes in the stationary growth phase at a parasite / macrophage ratio of 10:1 and incubated for 24 hours at 37 °C and 5% CO_2_ until promastigotes were phagocytized by macrophages. Then, extracellular parasites were removed by washing with cold PBS. Drug susceptibility of intracellular amastigotes was evaluated with a brief modification of the method previously described (9). This culture was incubated on ice for 10-15 minutes until infected macrophages were separated from the plates. The sample was stained by Giemsa and the percentage of infected cells and the number of amastigotes in each cell was assessed by light microscopy. Then, 100 µL of these cells were transferred to a new plate and incubated in the presence of five concentrations of *Artemisia sieberi* and artemisinin (5, 10, 25, 50, and 100 µg/mL) or in the absence of these drugs at 37 °C with 5% CO_2_ for 24, 48, and 72 hours. Finally, the plates were incubated on ice for 10-15 minutes, and then the samples were fixed with methanol and stained with Giemsa staining method, and the percentage of infection and IC_50_ was calculated through examination of the amastigotes inside the macrophage (200 macrophages per treatment) under light microscopy. The results were expressed as the infection index, which reflects the drug effect in preventing infection.

### 3.8. Statistical Analysis

The data were analyzed by SPSS version 16 with one-way ANOVA and Mann Whitney tests. The significant level is *P*-value less than 0.05. We also used Graph pad Prism software version 5.04.

## 4. Results

**Figure 1.**
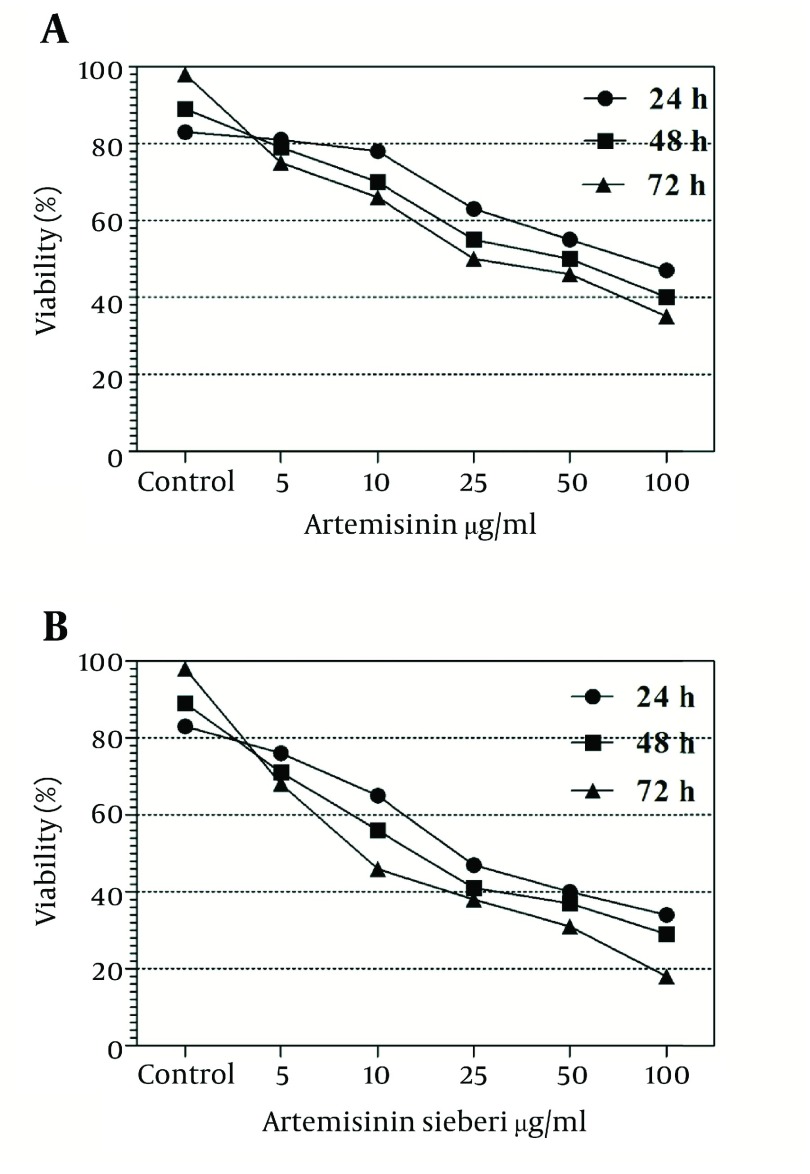
Viability of Promastigote in the Presence of Various Concentrations of Artemisinin and *Artemisia sieberi* in Comparison With the Test and Control Groups at Hours 24, 48, and 72

**Figure 2.**
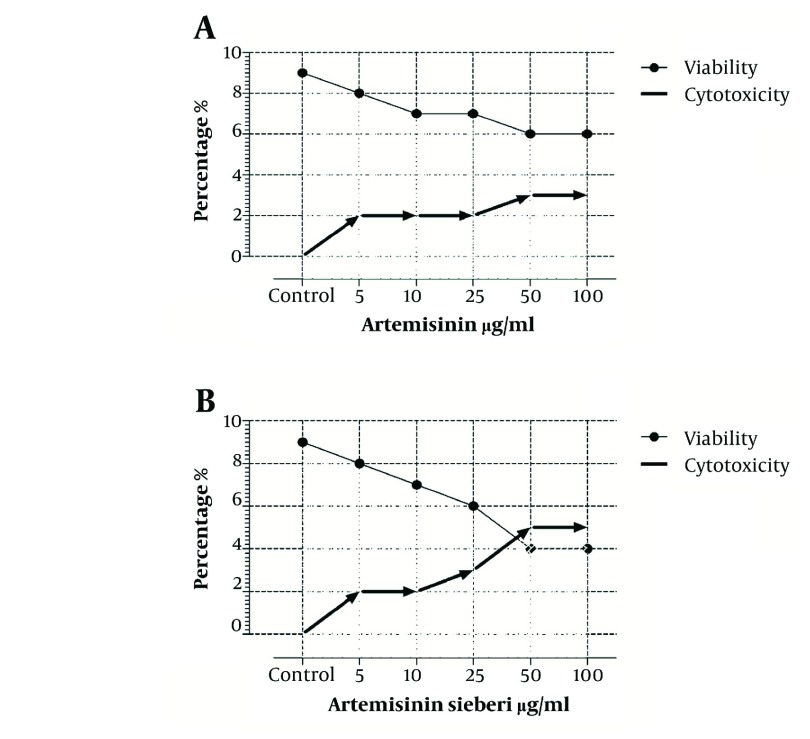
Effects of Artemisinin and *Artemisia sieberi* on Promastigotes of L. major Determined by MTT Method, and Comparing Them With the Results of the Control Group. Graphs A and B Belong to Artemisinin and *Artemisia sieberi*, Respectively

In the current study the effect of 5, 10, 25, 50, and 100 µg/mL concentrations of *Artemisia sieberi* aqueous extract and chemical artemisinin were evaluated under *in vitro* conditions on promastigotes and amastigotes of *L. major*. Furthermore, cytotoxic effects of these drugs were evaluated on uninfected macrophages. The results of the study demonstrated effectiveness of *Artemisia sieberi* and artemisinin on proliferation of promastigotes. However, *Artemisia sieberi* was more effective than chemical artemisinin in this respect. The growth curves of promastigotes are shown in Figure 1. In this figure, a dose - dependent growth inhibition was observed at 24, 48, and 72 hours after incubations. The IC_50_ (50% inhibition concentration of cell growth) was determined to be 25 µg/mL and 50 µg/mL for *Artemisia sieberi* and Artemisinin, respectively. Comparing the two drugs, *Artemisia sieberi* has a higher growth inhibitory effect on promastigotes. The effect of these drugs in the promastigote assay was confirmed by the MTT methods. Both drugs have cytotoxic effects in a dose-dependent manner. The IC_50_ values of *Artemisia sieberi* and artemisinin were determined as 25 and 50 µg/mL, respectively. According to the results, the aqueous extract of *Artemisia sieberi* could completely eliminate *L. major* promastigote and the results were confirmed by the MTT method. Results presented in Figures 1 and 2 showed a reduction in the parasite number as the dose of the drugs increased. Comparison of the effect of two drugs on promastigote that the number of promastigotes in the cultures treated with the plant extract it less than chemical drug.

**Figure 3.**
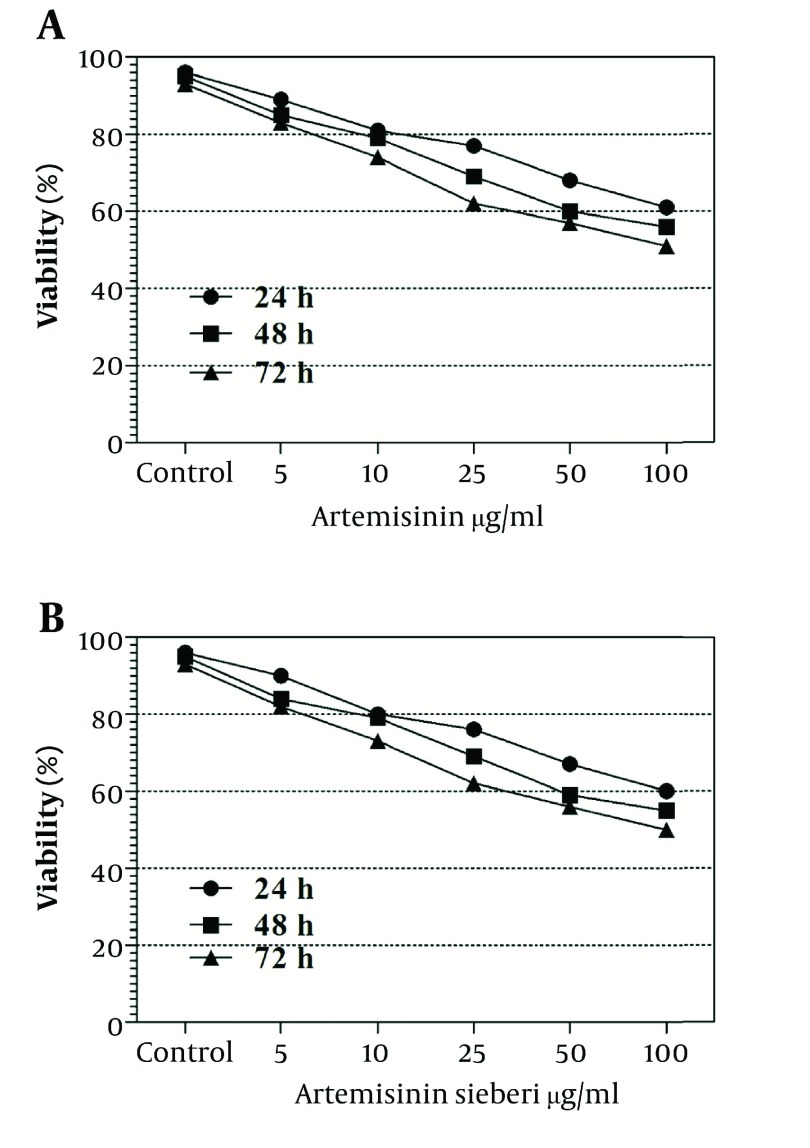
Viability of Uninfected Mouse Macrophages in the Presence of Artemisinin and *Artemisia sieberi* Under in Vitro Conditions in Comparison With the Control Group at Hours 24, 48, and 72

**Figure 4.**
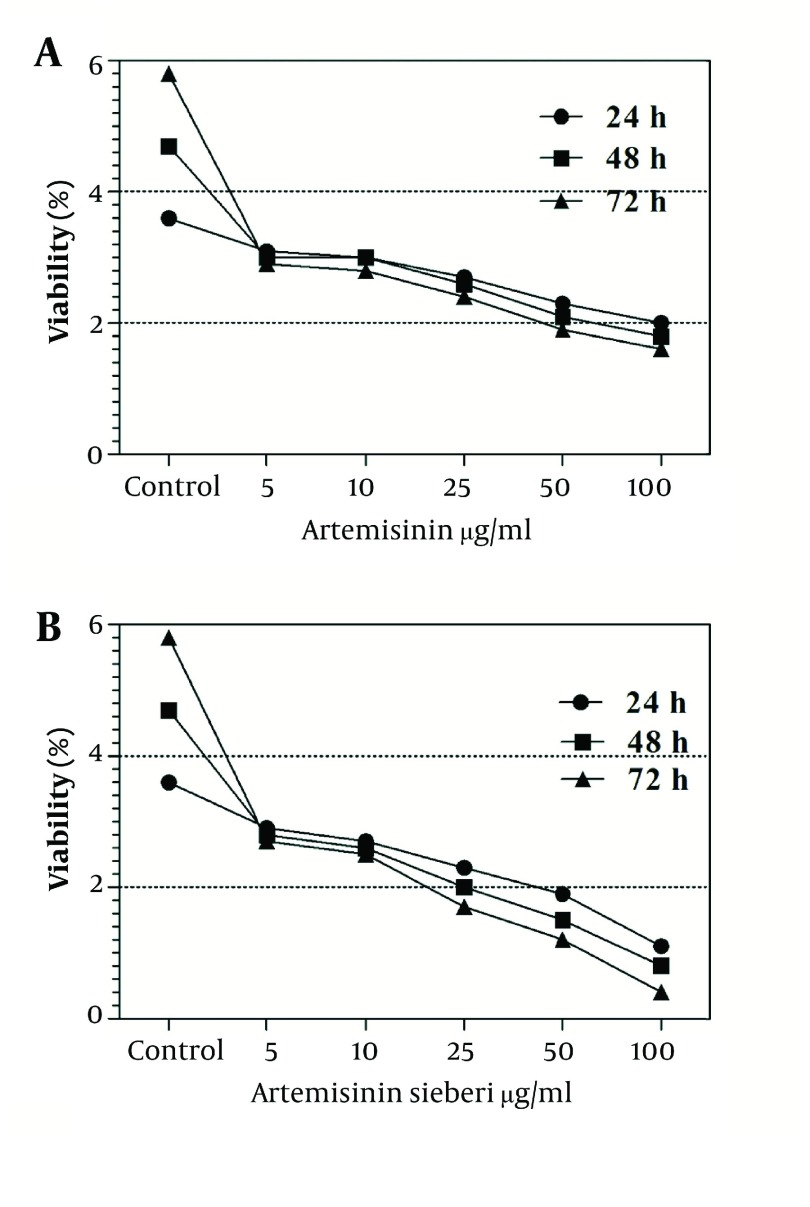
Viability of Mouse Macrophages Infected With Amastigotes of L. major in the Presence of Various Concentrations of Artemisinin and *Artemisia sieberi* Under in Vitro Conditions in Comparison With the Control Group at Hours 24, 48, and 72

The cytotoxic effect of seven concentrations of *Artemisia sieberi* on uninfected splenic macrophages of BALB/c mice was compared with the control cultures at hours 24, 48, and 72. In this respect, the results obtained for the two test cultures and the control cultures were not significantly different (*P* < 0.05). According to the results given in Figure 3, a decrease in the parasite number was observed as the drug doses were increased. It was observed that both *Artemisia sieberi* and Artemisinin have very low cytotoxic effect on uninfected and healthy macrophages (Figure 3). Compared with Artemisinin, *Artemisia sieberi* had a significantly lower cytotoxic effect on macrophages, as the IC_50_ values of *Artemisia sieberi* and artemisinin were 100 and 50 µg/mL, respectively. Both drugs were effective for elimination of amastigote from macrophages and decreasing the amastigote burden. In amastigote assay, it was observed that amastigote burden in macrophages was reduced as the dose of both drugs was increased. However, the rate was higher for *Artemisia sieberi* (Figure 4). Comparing the percentage of macrophages in *Artemisia sieberi* and artemisinin indicated that the cultures treated with the two drugs were significantly different from the control groups (*P* ≤ 0.05).

## 5. Discussion

Currently available drugs for cutaneous leishmaniasis are associated with many adverse effects. So, new strategies are required for treatment of leishmaniasis. The compounds evaluated in the study have effective properties against the parasite under *in vitro* conditions, rapidly arresting parasite metabolism in concentrations within the lower range, and killing the parasite more quickly than other antimalarial drugs (10). The effects of these drugs were evaluated on several organisms such as toxoplasma gondii and Pneumocystis carinii. Artemisinins are poorly effective at curing malaria, which is a phenomenon that is not well understood (10). *Artemisia sieberi* from Iran have been previously studied and the main components were found to be camphor, 8-cineole, camphene, and bomyl acetate (11, 12). In other studies, some *Artemisia sieberi* components showed antifungal effects. Other effects of this drug were reported to be anti-malarial, anti-viral, anti-tumor, anti-hemorrhagic, anti-coagulant, anti-oxidant, anti-hepatitis, and anti-ulcerogenic (11, 13-17). In Iran, some Artemisia species such as *Artemisia sieberi* are traditionally used for their various medicinal properties. For example, aerial parts of Artemisia species are used for their anti-viral effects (18, 19). In a previous study we have found that other plants such as *Alkanna tincturia* and *Peganum harmala* extracts have *in vitro* effects on *Leishmania major* (20). In the present study, our results revealed that both drugs have a dose-dependent anti-Leishmania activity. *Artemisia sieberi* is identified as an anti-Leishmania agent against promastigotes and amastigotes, while it showed lower toxicity toward uninfected macrophages, as the IC_50_ values of *Artemisia sieberi* for promastigote, amastigote, and macrophage were determined. The action mechanism of *Artemisia sieberi* against Leishmania is unknown. Considering the *in vitro* experiments, we found that amastigotes of *L. major* are very susceptible to *Artemisia sieberi*. *Artemisia sieberi* could decrease the number of amastigotes in macrophage cultures during the treatment. Medicinal plants and herbal drugs have some advantages over chemical drugs, including affordability, accessibility, and lower adverse effects. In summary, these satisfactorily results demonstrated that *Artemisia sieberi* can be a candidate agent for treatment of leishmaniasis in further studies. However, this requires further *in vitro* and *in vivo* studies. Moreover, evaluating the effect of the plant components on *L. major* should be determined. By analyzing the plant extract, its effective components can be used as a more effective drug.
